# Book Reviews

**Published:** 1799-03

**Authors:** 


### MISCELLANIES.

*An Experimental Dissertation on the Rhus Vernex, Rhus Radicans and Rhus Glabrum, commonly known in Pensylvania by the names of Poison-ash, Poison-vine, and common Sumach.* By THOMAS HORSFIELD of Bethlehem &c. *Philadelphia*, 8vo. 88 pp. 1798. Cist.

The botanical and chemical knowledge displayed in this performance, shews uncommon accuracy, and depth of research in the author. The plants described are all natives of New-York, as well as the Rhus Toxicodendron mentioned afterwards. Mr. Horsfield gives a minute description of the three species of Sumach, in the course of which he examines, whether the Rhus vernex, or Swamp Sumach be the varnish-tree of Japan, described by Kempfer, and gives his opinion that they are certainly the same plant.

The experiments detailed (p. 50-60) on the Rhus Glabrum, or common Sumach, tend to establish the following facts: That the saline concretion on the berries is a tartrite of pot-ash; that the gallic acid exists plentifully both in the capsules and leaves, but in the latter it is in a state more fit for economical purposes; that it may be advantageously applied to the dying of black, and the manufacture of ink, to prevent the necessity of importing foreign galls, and that its leaves may be substituted for oak-bark in tanning; discoveries which may be very valuable to the citizens of the American states. The leaves and storks of the common sumach are already much used in New York, in preparing sheep and goat skins, for what is called Morocco leather.

The remaining experiments related in this section, partly refer to the tartarous, acetous, and oxalic acids afforded by the rhus glabrum, with speculations on their convertibility into each other. After some further experiments and remarks on the black, yellow, and fawn colours, produced by the species of rhus, Mr. Horsfield gives an analysis of the vernex and radicans, and concludes with some observations on the medical properties of the latter, as an incitant and diuretic remedy of moderate efficacy, when infused in water, and taken internally: he recommends the external application of both, in certain stages of mania, melancholy, pulmonary consumption, epilepsy, palsy, and other chronic diseases, which have long resisted the efforts of very powerful medicines.

*Bibliotheque physico-economique, &c. The physico-economical Library, the 16th Year, containing Memoirs, Observations, and Practice in rural Economy, the most interesting new Discoveries, in the useful and amusing Arts; together with Descriptions of new Machines and Instruments employed in the same:* edited by Citizens PARMENTIER, and DEYEUX, 12mo. 400 pages. (2 liv. 12 sols.) *Paris.* Buisson,

We observe with pleasure the continuation of this useful work. Among a number of interesting articles on agriculture, rural and domestic economy, manufactures, sciences, and useful arts, the present volume contains the following:—"On the wheat with red ears, without beards, white grains, and a hollow stalk; by Tessier—on the danger of bathing in running water, by Bablot, and on Tokay wine by (the Right Honourable) Silvester Douglas.

*Memoires*

*Memoires presentant les Bases d'une nouvelle Théorie Physique et Chymique, &c. Memoirs presenting the Basis of a new Theory of Chemistry and Natural Philosophy, founded upon the Consideration of the essential Particles of compound Matter, and upon that of the three principal States of Fire in Nature: intended as an Exposition of the Work, intitled,* " *A Refutation of the Pneumatic Theory.*" By J. B. Lamark. 8vo. with ten Plates, exhibiting the new Classification of Animals and Minerals, and the natural order of Colours, (6 Liv.) *Paris.* Maradan.

These memoirs exhibit a *new theory of chemistry,* the principles of which, every where closely connected, and fruitful in conclusions, *appear* to furnish the solution of all known facts. The principal objects of the doctrine here explained, aims at determining the constituent parts of any compound body, as existing in the essential particles of the compound, and not in the mass of matter itself. The knowledge of such essential particles, and of the peculiarities relative thereto, forms the principal part of the studies and researches of the chemist, from which he can never deviate, without exposing himself to the errors that attend all reasonings which are supported only by arbitrary hypotheses.

This work also offers a *theory of fire,* altogether new, founded on an analysis of its principal existence in nature; its influence on bodies; the production of colour, combustion, or taste, according to their different states of combination. The Author farther endeavours to apply the general principles of physics, to the purpose of explaining the phenomena occurring in living bodies, as well as the origin and formation of minerals.

*Chemische and pharmaceutische Abhandlungen, &c.* " *Chemical and Pharmaceutical Essays, partly relative to medical jurisprudence, with a Treatise on Natural History. Translated from the Italian, and accompanied with remarks: by* I. A. Schmidt. M. D. 8vo. 264 pp. with two plates, *Leipzig* Schwickert.

These Essays possess considerable merit, on account of the sound reasoning, as well as the pertinent observations and experiments in which they abound. They well deserve to be translated. Our readers will be able in some degree, to judge of their value, from a general specification of their contents. 1. On the pernicious tendency of tinned copper vessels, used for culinary purposes. 2. On the necessity of keeping such vessels thoroughly clean, and the danger to families, and to society at large, from the neglect of this precautionary duty. 3. Remarks on the preparation of the dulcified spirit of nitre. 4. An analysis of the productions obtained by the decomposition of Sal-ammoniac. 5. Of the preparations of vitriolic æther, and the anodyne liquor of Hoffman. 6. An analytical account of the dulcified spirit of vitriol *(oleum vini.)* 7. An expeditious process of preparing phlogisticated alkali. 8. An historico-chemical essay on the former police of the City of Milan, with respect to the tan-works there established. 9. On the distilled oil of tartar. 10. A new process of preparing the kermes mineral. 11. On the properties of the oil of laurel, and its affinity to alkohol, æther, &c. 12. An improved Papin's digester, adapted to domestic and pharmaceutical uses; and, 13. an account of a remarkable stone, of an almost spherical shape, measuring three inches nine lines, in diameter, and weighing thirty-one ounces, found in the stomach of a horse, that died suddenly; and which concretion, from the imperfect analysis given by the author, we only learn, consisted of a great proportion of earth and oil.

Upon the whole, this collection is tolerably well written, and contains much interesting matter; but we regret that it is not so instructive as such a work might have been, had the author possessed a more accurate acquaintance with chemistry.

*Recueil des actes de la Société de Santé de Lyon, &c. The Transactions of the Society of Health at Lyons, from the first to the fifth year of the French Republic; or, Memoirs and Observations on different subjects of Surgery, Medicine, and Natural History.* (5 liv. 10 sols.) *Paris.* Perisse.

This collection, besides the light it throws on the general practice of medicine, contains a number of novel observations in that extensive science, as well as new facts relative to the animal economy, and natural History. There are annexed, two posthumous works of the celebrated Lecat, and observations on Surgery, by David, of Rouen.

---

*** *Want of room obliges us to defer the Review of the following newbooks until the next number.*

Memoirs of the Yellow Fever, which prevailed in Philadelphia and other Parts of the United States of America, in the summer and autumn of 1798: by William Currie, S.C. M.P. 8vo. 144 pp. Philadelphia. Dobson. 1798.

An outline of the history and cure of the fever, epidemic and contagious, more expressly the contagious fever of jails, ships, and hospitals, the concentrated endemic, vulgarly called the yellow fever of the West Indies; to which is added, an explanation of the principles of military discipline and œconomy, with a scheme of medical arrangement for armies, by Robert Jackson, M. D. 8vo. 396 pp. Edinburgh. Mundell, 1798.

An Account of the Plague which raged at Moscow, in 1771. by Charles De Mertens, M. D. trnslated from the French, with notes, 8vo. 122 pp. London. Rivington, 1799.

A New Inquiry into the suspension of vital action, in cases of Drowning and Suffocation. The third edition. To which are now added, A Preservative Plan, or Hints for the Preservation of Persons exposed to those Accidents which suddenly suspend or extinguish vital Action: by A. Fothergill, M. D. F. R. S. 8vo. 189 pp. and 43. price 4s. London. Rivingtons. N.B. The "*Preservative Plan.*" now first published, may be had separately, price 1s. 6d.

